# Social domains of poor mental health: A qualitative pilot study of community stakeholders’ understanding and demarcation of mental illness and its interpretations in rural Nigeria

**DOI:** 10.1002/hsr2.1922

**Published:** 2024-02-27

**Authors:** Saheed A. Lawal, Isaac A. Adedeji, Adesola Ogunniyi, David Henderson

**Affiliations:** ^1^ Department of Public Health, School of Public and Allied Health Babcock University Ilishan‐Remo Ogun State Nigeria; ^2^ Department of Gerontology Simon Fraser University British Columbia Canada; ^3^ Department of Medicine, College of Medicine University of Ibadan Ibadan Nigeria; ^4^ Department of Psychiatry, Boston University Medical Center Boston University Boston Massachusetts USA

**Keywords:** community stakeholders, mental health, mental illness, Ijebu‐Igbo Town, Nigeria, understanding and interpretations

## Abstract

**Background and Aims:**

Although previous studies on mental health/illness in Nigeria have explored knowledge and attitude of community members using quantitative approaches, few studies have engaged stakeholders within rural communities on the issue of mental illness using qualitative approaches. Community stakeholders play a critical role in influencing health behaviors. The objective of this pilot study was to explore community stakeholders’ understanding and demarcation of mental illness, and its interpretations in a rural Nigerian town. This is with the aim of shaping stakeholders understanding of people when they experience mental distress within the community.

**Methods:**

The study was conducted in Ijebu‐Igbo town of Ogun State in south‐west Nigeria. In‐depth interviews were conducted among two religious’ leaders: a Pastor and an Imam, a traditional healer, a medical doctor, and a registered nurse, and a focus group discussion was held in a church with members of its advisory committee.

**Results:**

The findings showed that community stakeholders gave multiple interpretations of mental illness and many attribute mental illness to spiritual attack, ancestral curse, anger of the gods, and personal affliction (Ogun‐Afowofa). This has been categorized as familial and individual attributes in this study which is part of the main themes derived. The study findings also show that the understanding of community members regarding the root causes of mental illness is somewhat vague based on their poor knowledge of mental illness. This is because of the various interpretations they gave to explain mental health is based on their cultural orientation, socialization, and belief system, and not based on any medical knowledge.

**Conclusion:**

This pilot study was conducted to justify the main study. There is therefore a need for health education interventions to enlighten and educate community stakeholders with requisite knowledge for better understanding and interpretation of mental illness. Also, through mental health education interventions, community members will gain clarity on what mental health is and what it is not.

## 1 INTRODUCTION

In many rural communities of Nigeria, there is a dearth of knowledge of the understanding and interpretation of mental illness. This is because certain socio‐cultural factors influence people's understanding of mental health and the possible causes of mental illness. In the wider Nigerian society, socio‐cultural factors play a central role in influencing the way people live and organize their daily living. As such, age‐old beliefs which are deeply rooted in traditions influence how people perceive those with mental illness. More so, the onset of mental illness has been associated with generational curses and evil proclamations in many rural communities. Changing these existing narratives will mean engaging community stakeholders that have some influence on other community members. Community stakeholders remain an important resource in promoting health and well‐being in rural areas. But, community stakeholders’ engagement in mental health/illness is understudied in many rural communities in Nigeria.

Previous studies on mental health in Nigeria have explored knowledge and attitude of community members to the various forms of mental illness through surveys,^1^, ^2^ while others have discussed the need for Nigeria's outdated mental health laws to be reformed.^3^, ^4^, ^5^ That is, the 1958 Lunacy Act is outdated and contains provisions that are not aligned with current international human rights standards.^6^ For example, chapter 112 section 24 on page 2300 of the Lunacy Act on the Escape of Lunatic states that “any person may arrest any lunatic who is escaping or who has escaped from lawful custody, and may return such lunatic into such custody.”^7^

Beyond the need for mental health reforms, some other studies and commentary articles have examined mental health in primary care settings,^8^ health workers’ knowledge of mental health,^9^ the beliefs of health workers about mental health^10^, and stigma and mental health in Nigeria with suggestions for law reform,^6^ community psychiatry care as an urgent need in Nigeria^8^, reforming Nigeria's outdated mental health laws^3^ and mental health in Nigeria is a neglected public health issue.^10^ Hence, the challenges of mental health persist because of poor societal attitudes toward mental illness, stigmatization toward mental ill persons, and reduced awareness on mental health.^3^, ^11^, ^12^ More so, the low priority accorded to mental health by the government.^13^

According to Suleiman^14^ the attention on mental health disorders is low in Nigeria, more so the awareness of Nigerians on mental health is considered poor, as misconceptions are expressed regarding mental health. Such misconceptions about mental health continue to thrive as an estimated 20%–30% of the population are said to suffer from one form of mental disorder or the other.^6^ Although Nigeria has a National Policy on Mental Health Services,^15^ the implementation of this policy is yet to translate to improving mental health services in the country. Hence, the increasing trend in the occurrence of mental health issues in Nigeria. However, community‐based qualitative studies on stakeholder's understanding and interpretations of mental illness in Nigeria are lacking, despite the major role of community stakeholders in global mental health care delivery.^16^, ^17^, ^18^, ^19^ More so, in the promotion of mental healthcare initiatives, community stakeholders are pivotal to its successful implementation.^20^, ^21^, ^22^ Therefore, engaging stakeholders in mental health research and other health research is important.^23^, ^24^, ^25^ For example, in Ijebu‐Igbo Town of south‐west Nigeria, the stakeholders are mainly adults and elderly people within the various communities. They play major roles in the maintenance and sustenance of the community. Therefore, what they know and how they interpret mental health is important. The aim of this study was to explore the perspectives of community stakeholders living in Ijebu‐Igbo Town of Ogun State, Nigeria on mental illness. More so, because culture play a major role in influencing the way people live and organize their lives in Nigeria, this study explored how culture influences the social interpretations of mental illness among community stakeholders.

## 2 MATERIALS AND METHODS

The study adopted the social constructionist tradition of qualitative inquiry using an exploratory phenomenological research design to elicit information. The social constructivist tradition aims to understand particular situations or phenomena, and to explore established knowledge and existing subjective realities.^26^ While the exploratory phenomenological research design was used to understand a given phenomenon's nature, by exploring the views and lived experience^27^ of community stakeholders that reside in study location. The study was conducted in Ogun State, located in the southwestern part of the country. The choice of the state is because it is home to the Neuropsychiatric Hospital (Aro) which is also a World Health Organization Collaboration Centre. Ijebu‐Igbo is a rural town in Ijebu‐North Local Government Area of Ogun State in Nigeria. The pilot study took place across five settlements/communities (Oke‐sopin, Ojowo, Ati‐kori, Oke‐Agbo, and Japara) in Ijebu‐Igbo town in Ogun State.^28^, ^29^ From each of the settlements/communities, a community stakeholder was selected and interviewed.

As seen in Table 1, a total of five in‐depth interviews (IDIs) were conducted among the community stakeholders each representing one of the five settlements/communities. Participants included a Pastor (Christian cleric), an Imam (Muslim cleric), a Doctor, a Nurse and a Traditional Healer. The age bracket of those interviewed was between ages 45 and 68 years old. A Focus Group Discussion (FGD) was held in a church with 12 members of its advisory committee between ages 35 and 75 years (Table 1). The 12 members of the advisory committee represent a different age category of community stakeholders and church members living in different communities in Ijebu‐Igbo town. Their roles include providing guidance and advise to the church central committee, planning church programs, and overseeing other church activities as leaders of various units. The occupational status of participants in the FGD varied. There were five traders (small businesses), two teachers, two retirees, a driver, a tailor, and a civil servant (government worker). The use of both the IDI and FGD for this study was to capture the views and experiences of different categories of community stakeholders. The FGD participants function as a group and not as individuals in carrying out their activities advisory committee members. This was the reason for conducting the FGD with this group of participants.

**Table 1:** Profile of participants.

S/N	Occupation	Age	Gender	Level of education	Duration of interview
	*In‐depth Interviews*				
1	Pastor	64	Male	Secondary O'level	42 min
2	Imam	68	Male	Secondary O'level	38 min
3	Doctor	46	Male	University first degree	58 min
4	Nurse	45	Female	University first degree	40 min
5	Traditional healer	57	Male	Primary school cert.	25 min
	*Focus group discussion*				1 h 10 min
1	Retiree	75	Male	Secondary O'level	
2	Teaching	59	Female	NCE	
3	Civil servant	58	Female	University first degree	
4	Business man	38	Male	University first degree	
5	Tailor	50	Female	Primary school cert.	
6	Driver	42	Male	Secondary O' level	
7	Retiree	72	Female	NCE	
8	Trader	60	Female	No formal education	
9	Trader	65	Male	No formal education	
10	Trader	56	Female	Primary school cert.	
11	Trader	60	Female	No formal education	
12	Teaching	36	Male	NCE	

The sampling procedure involved a non‐probability sampling method.^30^, ^31^ First was the purposive selection of Ijebu‐Igbo being a very large town in Ijebu‐North Local Government. Then participants for the FGD were drawn from the advisory committee of the largest church body in Ijebu‐Igbo; the advisory committee members were 23 in number. Only 12 willing participants of the advisory committee participated in the research. The IDI participants were stakeholders who represented the five settlements/communities in Ijebu‐Igbo town. The reason for selecting these participants is because their knowledge, attitudes, awareness, and perception of mental health and illness are important^32^ to influence community members. Also, the effective engagement of community stakeholders in mental health care is empowering for community members.^23^, ^24^ The sample selection included religious leaders (Iman; Pastor) who provide spiritual guidance to community members. The pastor is the head in one of the large churches in Ijebu‐Igbo. The Imam is the head of the oldest mosque in the town. Health workers such as doctors and nurses were qualified to take part in the research. The Doctor is the consultant in‐charge of one of the oldest private hospitals in the town and the nurse also works in a community‐approved primary health center. The doctor provides essential medical services such as diagnosis, treatment, and preventive care, while the nurse gives patients their medications, monitor patients' vital signs, and documents patients’ condition with other duties assigned to them. Healthcare delivery in Nigeria is complex and comprises several providers^33^, ^34^, ^35^, ^36^; therefore, an elderly traditional healer with many years of experience was also eligible to participate in the study. Such a traditional healer is knowledgeable in the use of herbs for treatment of various diseases and ailments. They “play an essential role in the delivery of primary health care to local people as they treat people in resource poor settings.”^37^ The selection of these individuals for the study is deliberate because of their importance in healthcare delivery within rural settings.

The eligibility criteria for selecting each of the participants were followed. The inclusion criteria were used to ensure that the religious leaders were the actual church or mosque representative. Such a person must have been appointed or nominated by the religious body into such a position, while the community healthcare workers (Doctor or Nurse) were a certified doctor and nurse who have been working in the community health center/post or in a private hospital/clinic for more than 3 years. For the traditional healer, such a person must be a folk healer or spiritual healer who is known by the people in the community. For those eligible to participate in the FGD, they must be either a male or a female who resides in the community and has lived there for over 5 years. The reason is because such persons who have lived in the community for this long are considered to be more knowledgeable about the way of life in the community. He or she must be above 35 years of age and an advisory member of the church committee. In addition, the rationale for the age condition of participants in the FGD is simply because they are older adults and not young persons. These elderly people constitute the major stakeholders in Ijebu‐Igbo town. They have lived long in the town and possess some influence. A religious leader who is not the ordained church or mosque representative is excluded. That is, a person who has not been appointed or nominated by the religious body into the position they occupy was also excluded. Doctors or nurses who have not worked more than 3 years in Ijebu‐Igbo town were not eligible for the study.

The questions asked in the pilot study explored the social construction of mental health at the community level. The guide had 13 questions and 5 probe questions. Questions such as what is your understanding of mental illness? What are the sources of mental illness? What the known and unknown causes of mental illness in the community? What are the various types of mental illness known to you? Does culture affects the way people view mental illness? were in the IDI and FGD guide. The interviews lasted for about 25–60 min. While the FGD that was conducted had a similar number of questions and had both male and female participants. The FGD lasted 1 h 10 min.

The data were analyzed as appropriate in line with the focus of this study. First, it was transcribed verbatim to Yoruba the original language in which two of the interviews (traditional healer and Imam (Muslim cleric) and the FGDs were conducted. Afterward, the two interviews and one FGD were then translated into conventional English (without distorting the lingua, expressions, and context of the discussion). This social process of the translation considered the intonation and pauses expressed by participants during the interview sessions. For example, some pauses were used to mean concern or displeasure on why the socio‐cultural factors continue to shape people's understanding and interpretation of mental illness. Words that did not have a direct English interpretation were left in the original Yoruba language but circled. These words could only be described to express their original meaning. All discussions with the interviewees and FGD participants were tape‐recorded. Each of the participants’ consent was first obtained before interviewing commenced. Three of the interviews (doctor, nurse, and pastor) were conducted in English language. These were also transcribed verbatim.

Second, a coding scheme (codebook) was developed for the transcripts based on issues identified during the data analysis after the data transcription. The codebook provides a guide on how to code qualitative data. Codebook provides a stable frame for the dynamic analysis of textual data.^38^ The codes developed in this study were harmonized to ensure consistency before being applied to the data. After which the thematic content analysis was used to break down the data into major themes (parent code) and sub‐themes (child code). Some of the parent themes include causes of mental health, types, treatment, education, and stigma, while some of the sub‐themes are self‐affliction, alcoholism, drug abuse, evil curse, ancestral effect, neurosis, schizophrenia, and mad people. Each of the major themes was derived using an iterative process to identify its occurrence in the entire data points. Following this, key themes or constructs were identified from the transcripts and matrices developed to discern patterns and relationships among themes.^39^ Themes that emerged from the data were shared with the study participants for validation before they were reported on and used in the study.

Third, Inter‐coder reliability also known as inter‐coder agreement/inter‐rater reliability^40^, ^41^ was used in this study by two independent coders to evaluate the data from the field during the analysis. Each of the coders worked on the transcripts independently to derive the parent codes and child codes. Afterward, each of their codes were compared and harmonized to derive a uniform code that formed the codebook. The general codebook guided the content analysis of the entire data sets. The data are presented in quotation form to support each major theme derived from the transcripts.

### 2.1 Ethical considerations

Ethics approval was obtained from the Social Sciences and Humanities Ethics Committee/University of Ibadan, Ibadan, Nigeria. The IRB number is UI/SSHREC/2018/0037. Verbal consent and a signed informed consent form were obtained from the study participants. The identities of participants remained anonymous as each was assigned a number. All identifiers were removed and stored in an encrypted data file that is password protected.

## 3 RESULTS

The following quotations and interpretations are used to describe community stakeholder's understanding and demarcation of the concept of mental health, and their perspective on the root causes vis‐à‐vis their interpretations of mental illness.

### 3.1 Familial and individual attributes as basis for the social domains of poor mental health

The knowledge of the causes of poor mental health as presented by stakeholders in rural communities was in alignment with familial and individual attributes derived from the study. Knowledge of poor mental health originated from spiritual and cultural interpretations and evolved into stigmatizing contexts which consisted of misconceptions negating biological and psychosocial explanations. The misconceptions associated with poor mental health include the idea that it is an inherited and generational health condition which may be biological or arising from a curse or some form of enchantment by evil‐doers. In terms of the individual attributes, at the intersection of familial and individualistic explanations, is the shared belief that parents can curse a child with mental illness. Importantly, poor mental health was conceived as emanating from spiritual (curses) and behavioral self‐affliction (outcome of illicit use of substances).

A participant presented both the familial and individualistic explanation of the causes of poor mental health. It was noted that:

You see about mental illness, so many things are the causes, firstly; generational illness causes it, maybe you find someone that is afflicted of that illness in that family. Secondly; self‐affliction like drinking illicit drinks, weed, cocaine, that's two, the third one is (may God not let us offend evil‐doers) they can curse someone, so that what I see about that. (Participant One, FGD, Ijebu‐Igbo)

In reinforcing the individualistic perspective to the onset of poor mental health, a Nurse explained further:

…some of the people will say it is because of a curse, that someone has used a spell on them (mad person/mental ill person). But majority believe in this town is that mental illness is being caused by either incantations or curse from one god or the other. (Nurse, 45, Ijebu‐Igbo)

### 3.2 Individual characteristics as the causes of mental illness

Following the demarcations of familial and individual attributes as the basis for the occurrence, this study considers the factors associated with such manifestations. Various factors are identified as the causes or sources of mental illness within Ijebu‐Igbo town. The demographic description of persons with mental illness in the community focused on young people. On an individualistic basis, some believed mental illness is self‐inflicted especially when a person consumes hard drugs and substances that affect normal brain functions. Many young persons in the community are known for consuming hard drugs such as tramadol, codeine, cough syrup, and cannabis. As such, the narratives were more specific about drug use as fundamentally responsible for mental illness. Results showed that for individualistic causes, there are two clear directions. First is the individualistic mental illness arising from behavioral patterns which include the use of hard drugs and substance. Second is the spiritual/mystic perspective that is linked with existing cultural beliefs. With regard to the behavioral factors, the use of cannabis (weed) (colloquially referred to as ‘igbo’ in the Yoruba language), tramadol and codeine were listed. The individualized onset of mental illness was also associated with the heavy use of alcohol. In providing a spiritual dimension to the individualistic cause of mental illness, a nurse provided an explanation regarding the role of stress, incantations, and the anger of the gods toward an individual.

This finding is supported by another FGD participant's view that:

Most of those that you see that are mad, it's not that they were cursed but rather their drinking of harmful things is too much, they will smoke weed and all sorts of things like taking tramadol and codeine. Kids that are not up to [sic] age that will be drinking heavy alcohol and doing all manner of things… (Participant Seven, FGD, Ijebu‐Igbo)

Another participant from the FGD noted that:

Back in the olden days, curse is what they [sic] give to them but nowadays self‐caused affliction is what they do, a child that is not of age, will be smoking weed and will be saying that “what are you, what can you do” some will just throw down their clothes and will be going stark naked…. (Participant Ten, FGD, Ijebu‐Igbo)

But majority, the belief in this town is that mental illness is being caused by stress, incantations and chants, or the anger of one god upon a person and their family…. (Nurse, 45, Ijebu‐Igbo)

### 3.3 Familial attributes/factors

Results from the study describe the familial explanation for mental illness in the community. The onset of mental illness has been associated with generational curses and evil proclamations. Mental illness has been hinged on age‐old beliefs which are deeply rooted in tradition. In addition, familial factors have been linked to the heritability of mental illness. Narratives from the study implied that community members perceive a spiritual and biological transmission of mental illness along generational lines. This is evident in their allusions to biological, generational, and spiritual explanations regarding the onset of poor mental health in the community.

Some FGD participants stated that:

…Its from a family disease that is inherited. So, there is nothing they can do about it except they should just take care of them (person with mental illness). (Participant Four, FGD, Ijebu‐Igbo)

…you see about that mental illness, so many things are the causes, firstly; generational illness causes it, maybe you find someone that is suffering from it in that family, that is one…. (Participant One, FGD, Ijebu‐Igbo)

…about mental illness, number one, inheritance in the family. This usually occurs when it's about someone having mental illness…. (Participant Two, FGD, Ijebu‐Igbo).

### 3.4 Common mental illnesses

In Ijebu‐Igbo town, stakeholders in community health identified the common types of mental illnesses in the community. Doctors, nurses, and traditional healers who diagnose and provide care to cases of mental illness provided narratives about the occurrence of conditions like schizophrenia, psychosis, and depression. These findings align with the preponderance of substance abuse and the prevailing manifestation of mental illness among young persons living in the community.

A participant was of the view that:

We have neurosis, we have schizophrenia…they are grouped according to their manifestations. That is why we have depression too. (Nurse, 45, Ijebu‐Igbo)

Mental illness in the community varies. It is a spectrum, you could have things like depression, you could have anxiety, you could have psychosis, you could have…it is a spectrum, there is a whole range of things in there. (Doctor, 46, Ijebu‐Igbo)

A different participant stated that:

…It usually comes that one can suddenly get extreme high temperatures that can turn into mental illness. You see that high temperature can led to deafness that can in turn lead to mental illness…You know these mental ill persons we are talking about, there are “corporate madness, and there are different types that divides in different ways…. (Traditional healer, 57, Ijebu‐Igbo)

### 3.5 Socio‐cultural construction of mental illness experiences

In Nigeria, socio‐cultural factors play a central role in influencing the way people live and organize their daily living. This is because culture is a complex whole that is comprised of beliefs, customs, morals, values, traditions, language, religion, and rules of behavior within any given community. Hence, in the rural settings of south‐west Nigeria, people often conceive and define their social reality based on their cultural orientation. This influences their knowledge, understanding, attitude and perceptions of mental illness.

A participant stated that:

There are some spirits that directs them, some will hear the voice in their ear to do this and that, they will hear, and no one will hear what they hear. (Traditional Healer, 57, Ijebu‐Igbo)

Due to people's beliefs, the way they tend to perceive mental illness varies. Another participant expressed his opinion that:

In the community, on the streets you will surely see them, you surely know them because they, they will, you can see them differently, through their hair, through their clothes, through their erh… this thing. You just erh… when you sighted them you know this one is another insane person. (Pastor, 64, Ijebu‐Igbo)

An in‐depth interview response revealed that:

Well you know those mental ill people, once they go mad, they don't know what they are doing anymore, some will be hearing voices that tells them to do this and that and they don't know… (Traditional Healer, 57, Ijebu‐Igbo).

## 4 DISCUSSION

**Figure 1:**
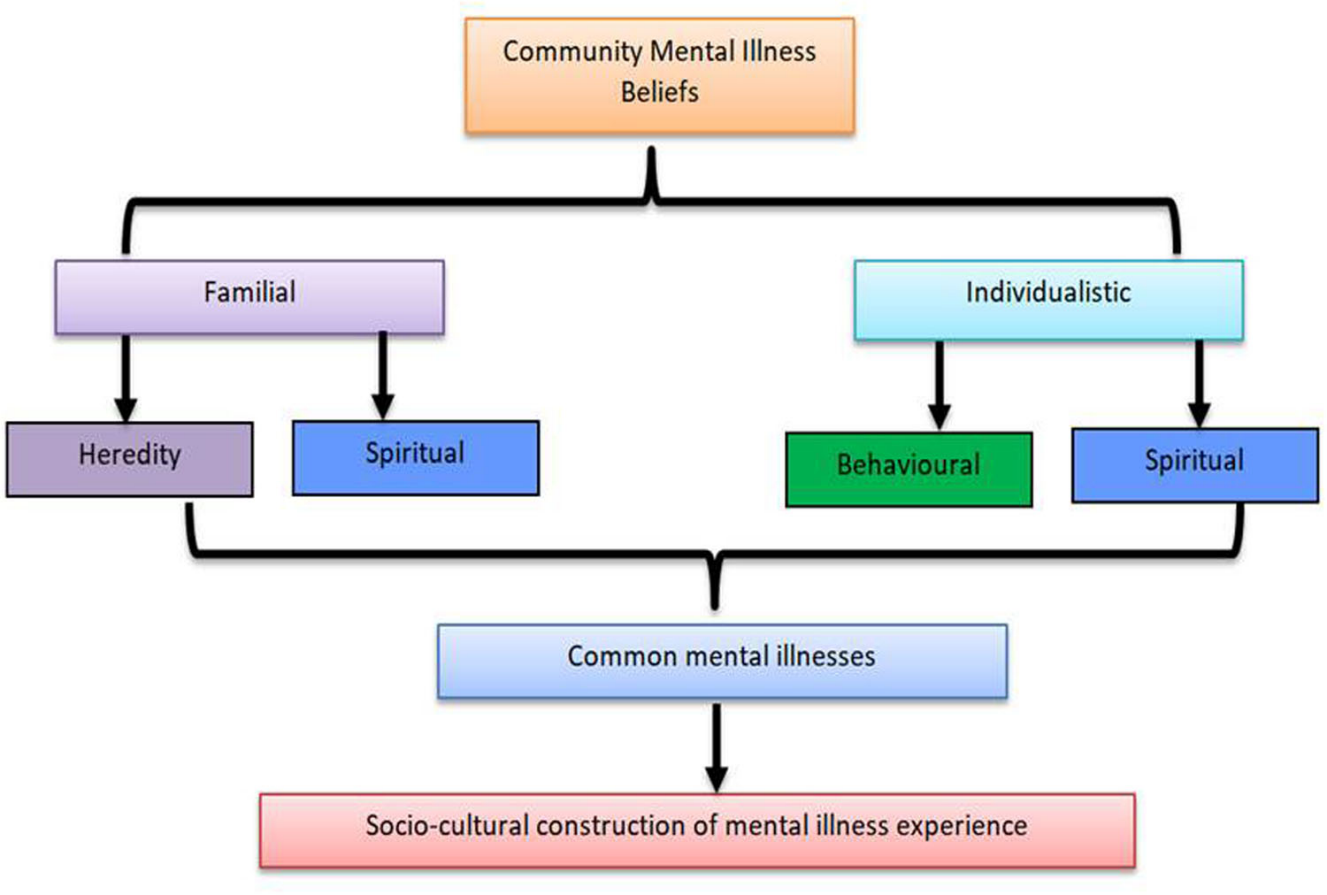
Social domains of poor mental health.

This pilot study explored community stakeholder's understanding and the demarcation of mental illness and its interpretations in a rural Nigerian town. Findings show that among community stakeholders their understanding, interpretations, and demarcation of mental illness are based on familial and individualistic attributes as seen in Figure 1. For the community stakeholders, the familial include issues such as heredity and spiritual causes of common mental illness in Ijebu‐Igbo town. While for some stakeholders, the individualistic issues are drawn from behavioral and spiritual causes. Hence, common mental illness beliefs are demarcated based on these two broad interpretations of mental illness as reported in the study. This forms the social construction of mental illness experience in Ijebu‐Igbo town.

### 4.1 Community stakeholders’ understanding of mental illness and the cause of it

In Ijebu‐Igbo town, people say different things about mental illness making it difficult to understand and capture the community thoughts. This is further aggravated by the stigma associated with mental illness in Ijebu‐Igbo. According to Sheikh et al.,^42^ stigma affects the effective delivery of mental health interventions. In Ijebu‐Igbo town, the people's understanding of mental health is drawn from family background and lineage. For a person to become mentally sick, it means it runs in their families. As such, people's understanding of mental health is linked with a generational curse that runs in a family. These generational curses are unknown but once reports have been heard in the community that a person in the history of a family was afflicted, then others are susceptible to becoming mentally ill. This is widely believed. Although there are no scientific explanations for these claims, still people attribute a mental health challenge to lineage and family background. This represents the understanding of stakeholders in Ijebu‐Igbo town. Generational curses have been identified as one of the causes of mental illness. Other studies such as Kishore and colleagues^43^, ^44^, ^45^ have all reported on generational curses as being a major cause of mental illness, especially among people in rural communities.

Studies^43^, ^44^ in Nigeria and beyond have reported that mental illness is caused by various factors that are social, cultural, economic, or environmental in nature. Also, Jacob^46^ and Mirzaei et al.^47^ reported that stress, depression, and anxiety are common causes of mental illness in a population. This study reported a similar trend but differed as the findings show that mental illness is now being self‐inflicted by the person with mental illness. That is, this study asserted that mental illness in the community is sometimes self‐inflicted. Self‐infliction includes the use and consumption of alcohol, hard drugs, and substance. Only a few studies in Nigeria have linked hard drugs consumption with mental illness. Although this link is well established by Adrian and Barry^48^ that mental health problems can be associated with the use/misuse of drugs and alcohol. Persons without a history of mental illness in their families who misuse drugs and abuse alcohol are now susceptible to developing physical and mental health problems, as witnessed in Ijebu‐Igbo.

The findings of this study indicate significant emphasis on the regular consumption of local alcoholic drinks of which the volume is often not determined or specific. Hence, these local drinks when consumed too much tend to affect the user and often cause them to lose touch of what is real and what is not. In their study, Otim et al.^49^ reported a similar finding on the negative effect of high levels of consumption of locally brewed gins and spirits. They stated that these alcoholic drinks do not have a scientific valid measurement on how much must be consumed per time, their composition, or which of their contents are injurious to human health. Beyond the consumption of locally brewed gins and alcohol, this study found out that in Ijebu‐Igbo town, the belief that mental illness is attributed to the anger of the gods is widespread. Also, when an evil proclamation is made upon a person, such a person can develop mental illness. People believe that those who suffer from any form of mental illness do so as a result of them being punished by the gods of the land or their ancestors. For example, a man may be cursed for desecrating the native shrine of the gods, or by a witch doctor or a very wicked person such as the indigenous medicine man (*Onisegun/Babalawo*) who is sometimes referred to as the local herbalist/traditional healer. He or she may be cursed by an elder in the community whose connection to the gods is quite powerful. All these occurrences are considered in the community as possible reasons why a person may become mentally ill.

### 4.2 On the concept of mental illness and mental health

In Ijebu‐Igbo town, people with specific knowledge of mental illness include the traditional healers, elders, doctors, clergies, and nurses. These people have some understanding of what mental illness means. That is, their interpretation of mental health is linked with the imminent manifestations that are seen by all members of the community as found in this study. According to Okpalauwaekwe et al.,^2^ mental illness in Nigeria is commonly identified when people see those affected behavior abnormally and manifesting conditions such as mild psychosis or schizophrenia. In the study area, other manifestations of mental illness occur in the forms of depression and anxiety. The people across communities in Ijebu‐Igbo town know what mental illness is because they can identify people who have a certain type of mental illness, although they have some myths, beliefs, and realities associated with mental illness and common psychiatric disorder across Africa.^50^ Yet their understanding of psychiatric disorders in Ijebu‐Igbo has led to the many different cultural interpretations of the various dimensions of mental illness. Hence, there are several descriptions of the various types or forms of mental illness in the communities. These many interpretations also lead to confusion in explaining the specific types of mental illness that exist within the communities. Although previous studies such as Ekwueme and Aghaji^9^ and Ewhrudjakpor^10^ have reported on the knowledge and attitude of health workers on mental illness. While Gureje et al.^1^ reported on community knowledge and attitude of mental illness in the south‐west region in Nigeria using a quantitative approach, but community‐based qualitative studies at the rural level are limited. In addition, past studies on mental illness in Nigerian communities in the past four decades show that people do not have enough knowledge of mental illness.^1^, ^2^ This in turn affects people's attitudes toward those who are mentally ill in their communities.

### 4.3 Interpretation and demarcation of mental illness

In Ijebu‐Igbo, people have more than one way of interpreting mental illness. Some base it on their experience and others do not. In the rural settings of south‐west Nigeria, culture plays a major role in influencing people's behavior. Culture is a product of people's philosophies, ideas, customs, norms, principles, perceptions, beliefs, attitudes, and so forth. A people's perception of health and illness is defined based on their culture.^51^ The people of the Ijebu‐Igbo town believe that people who suffer from mental illness are being controlled by certain unseen spirits. These spirits control them daily. Many of these spirits remain unseen to the natural human eyes. These spirits direct the daily affairs of people who are mentally ill; they take charge of their minds and direct them on what to do and what not to do. They simply assume control and do as they please. These spirits tell mentally ill people what they want from them and guide them on how to go about the task. However, their voices are not heard by other members of the community, only by the person who is mentally ill. According to scholars who study traditional religions and divinities, and argue that the natural things of life can be manipulated from other realms beyond what is seen by all persons, some spirits bless people, bringing them good fortune, while some others punish people for their wrongdoing in the community. So, for those suffering from mental illness, the spirits are working against them. More so, the Yoruba people believe that mental illness cannot be permanently cured^52^ because these spirits never go away completely. There is therefore the need for a national mental health education program that is culture‐sensitive drawn from the first Mental Health Act/Law postindependence of 1960, which was signed into Law in January 2023 by the Nigerian President. This is because the National Mental Health Act 2021 which is a paradigm shift^53^ from the previous Act aims at addressing existing mental health challenges in Nigeria. The new Act holds the potential to incorporate mental health into primary health care which will invariably “improve accessibility, affordability, and cost effectiveness, while advancing human rights”^54^ based on effective implementation. A national mental health education program drawn from the new Act can provide Nigerians with the right knowledge and understanding of mental health issues.

### 4.4 Limitations

This pilot study was not without limitations. First, the pilot sample is small. This is because only a few of the community stakeholders approached were willing to participate in the study. Also, the aim of the pilot study was to gain some insights into the way community stakeholders interpreted and demarcated mental illness. The findings of the pilot study were used to refine the interview guide of the main study. Second, the study was only limited to five stakeholders from the five settlements/communities in the whole of Ijebu‐Igbo town being the second largest in Ogun State.

## 5 CONCLUSIONS

Engaging community stakeholders in any community‐based mental health intervention in rural areas is pertinent to its success. Therefore, having some insight into their understanding and demarcation of mental illness and its interpretation is important. This pilot study reports on the varied perspectives of community stakeholders on the social domains of poor mental health in Ijebu‐Igbo Town of Ogun State Nigeria. Community stakeholders’ understanding is based on the familial and individualistic attributes which form people's misconceptions leading to different interpretations and demarcations of mental illness. Hence, there is a need to educate and enlighten people in Ijebu‐Igbo on the nature and complexities of mental health in the 21st century. If community stakeholders continue to lack proper understanding of mental health, such communities will continue to be affected.

## AUTHOR CONTRIBUTIONS

**Saheed A. Lawal**: Conceptualization; formal analysis; investigation; methodology; validation; visualization; writing—original draft; writing—review and editing. **Isaac A. Adedeji**: Funding acquisition; methodology; project administration; validation; writing—review and editing. **Adesola Ogunniyi**: Funding acquisition; methodology; project administration; resources; supervision; validation; writing—review and editing. **David Henderson**: Funding acquisition; methodology; project administration; resources; supervision; validation; writing—review and editing.

## CONFLICT OF INTEREST STATEMENT

The authors declare no conflict of interest.

## TRANSPARENCY STATEMENT

The lead author Saheed A. Lawal affirms that this manuscript is an honest, accurate, and transparent account of the study being reported; that no important aspects of the study have been omitted; and that any discrepancies from the study as planned (and, if relevant, registered) have been explained.

## Data Availability

All data analyzed for and presented in this article are from the in‐depth interview and focus group discussions transcripts. The data that support the findings of this study are available on request from the corresponding author. The data are not publicly available due to privacy or ethical restrictions.
